# Deep learning-enabled mobile application for efficient and robust herb image recognition

**DOI:** 10.1038/s41598-022-10449-9

**Published:** 2022-04-21

**Authors:** Xin Sun, Huinan Qian, Yiliang Xiong, Yingli Zhu, Zhaohan Huang, Feng Yang

**Affiliations:** 1 School of Traditional Chinese Medicine, Beijing University of Chinese Medicine, Beijing, 100029 China; 2 School of Traditional Chinese Classics, Beijing University of Chinese Medicine, Beijing, 100029 China; 3 School of Chinese Medicine, Beijing University of Chinese Medicine, Beijing, 100029 China

**Keywords:** Machine learning, Computational models, Classification and taxonomy

## Abstract

With the increasing popularity of herbal medicine, high standards of the high quality control of herbs becomes a necessity, with the herb recognition as one of the great challenges. Due to the complicated processing procedure of the herbs, methods of manual recognition that require chemical materials and expert knowledge, such as fingerprint and experience, have been used. Automatic methods can partially alleviate the problem by deep learning based herb image recognition, but most studies require powerful and expensive computation hardware, which is not friendly to resource-limited settings. In this paper, we introduce a deep learning-enabled mobile application which can run entirely on common low-cost smartphones for efficient and robust herb image recognition with a quite competitive recognition accuracy in resource-limited situations. We hope this application can make contributions to the increasing accessibility of herbal medicine worldwide.

## Introduction

As the primary healthcare system for about $80\%$ of the world’s population, especially in the developing countries^1^ and also an increase in developed countries^2^ such as France and Germany, herbal medicine has gained cumulative popularity in today’s medical practice due to its cultural acceptability, availability, affordability, efficacy and safety claims^3^.

Herbal medicine, as defined by World Health Organization (WHO), is the practice which includes herbs, herbal materials, herbal preparations and herbal products containing active ingredients parts of plants, or other plant materials, or combinations^3^. Among the four forms of herbal medicine, the herbs are basic and distinctly important as they are the source and main component of other forms. Therefore, the quality control of the herbs becomes a key problem, which will directly influence herbal products in their acceptability and efficacy^4–6^. Meanwhile, the worldwide upsurge of applying herbal medicine is calling for high standards for the herb quality, of which the herb recognition telling herb category and providing herb quality rating is the key step.

Herb recognition faces great challenges. Firstly, the herb quality is closely related to many factors, such as the temperature, use of fresh plants, light exposure, nutrients, water availability, period and time of harvest, method of harvesting, drying, packing, storage and transportation. Secondly, herbs are usually complicatedly processed from plants, and their appearance will be much different from their original forms. Thirdly, there are thousands of common herbs worldwide, which makes it difficult and time-consuming to recognize all the categories. Over the past decades, two primary solutions have been proposed, namely, the manual recognition and automatic recognition.

**Figure 1:**
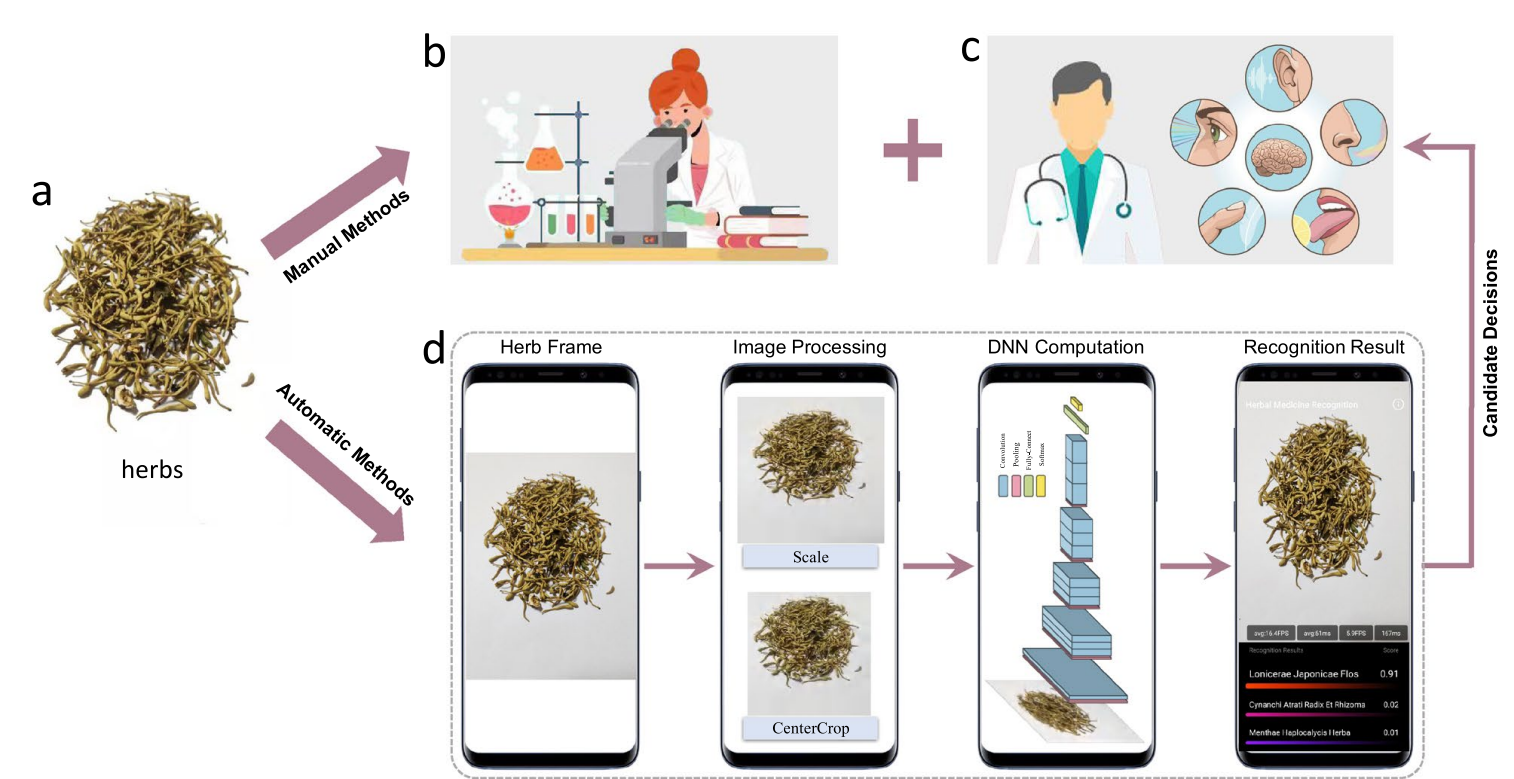
An illustration on how the deep learning-driven mobile application works for herb recognition. Given some herbs (**a**), manual methods use fingerprint technology (**b**) or expert experience (**c**) for recognition. We consider the automatic way to recognize the herb image with a deep learning method on a single smartphone (**d**), which can provide candidate decisions to speed up the manual recognition.

In manual recognition, one popular method is the herb fingerprint technology (Fig. 1b). It extracts the biological fingerprint of herbs by complicated chemical steps, which include herb splitting, Gas Chromatograph-Mass Spectrometer-computer (GC-MS) analysis and fingerprint generating, and then the fingerprint is compared to the template of each category based on their similarity^7,8^. Because of its reliability and accuracy in herb recognition, it has been accepted as a standard pipeline worldwide^3^. Another solution of manual recognition is the experience based method, wherein professionals use their long-time experiences by looking, smelling, tasting, touching and hearing (Fig. 1c). However, manual recognition has its limitations. Firstly, it cannot work without professionals with rich knowledge and experience, or chemical materials and devices that may not be available for resource-limited settings. Secondly, it will cost too much resources and time to recognize thousands of herbs manually.

In order to alleviate these drawbacks of manual recognition, automatic recognition has been introduced based on the idea that utilizing herb images and giving quick candidate categories to assist the manual recognition to speed up making final decisions (Fig. 1c). In conventional approaches differentiating herbs according to color, shape and texture, the accuracy cannot be satisfied due to their low-level appearance property^9–11^. Recently, inspired by the development of image recognition in computer vision^12–16^ and medical areas^17–23^, deep learning based methods have shown great improvements^24–29^, which makes it feasible to provide candidate decisions with satisfied accuracy. Most studies have used the Deep Neural Network (DNN), which combines with powerful and expensive computation hardware to support the computation. This combination is commonly used in hospitals and laboratories, and it aims towards a high degree of system automation and high system stability.

However, due to the high price of the hardware, it is not suited to dispensaries or hospitals in resource-limited settings. Simply reducing the hardware requirements is not feasible, as the DNNs are usually large and heavily-designed, costing too much computation. Therefore, the key of our application design is to obtain an efficient and accurate small DNN that can be deployed in resource-limited devices. One intuitive idea is to use a small DNN directly. However, the recognition ability will be weakened due to its fewer parameters, which will lead to the large drop of accuracy, even though the efficiency can be largely improved. As a result, we shifted into another direction: can we compress the large DNN into a small one? Though cutting a small portion of parameters of the large DNN can work in some cases^30–32^, it still has three main limitations. Firstly, cutting the parameters cannot change the inner network structure, but can only make the network smaller, which may not achieve the expected efficiency in low-cost situations. Secondly, due to the huge gap between the large and small DNN in the number of parameters, cutting a large portion of parameters will lead to the obvious drop of recognition accuracy. Thirdly, the large gap also makes it hard to give accurate control on how much to cut to the target level. To meet these challenges, we propose a three-step network compression algorithm with each step corresponding to each limitation in order. Some recent studies show that specific network structures are appropriate for low-cost situations^33,34^, and the DNN with these structures can obtain satisfying accuracy with fewer parameters and higher efficiency, which matches our motivation perfectly. Inspired by this, our first motivation is to use a separate small DNN with these efficient structures. Then, given the fact that cutting parameters cannot be done with different network structures, our second motivation is to transfer the recognition ability from the large DNN to the small one to keep accuracy. Finally, within the same structure, our third motivation is to give precise control of cutting the small DNN to achieve the best trade-off between accuracy and efficiency.

In this paper, we propose a novel deep learning based network compression algorithm to compress the heavily-designed DNN to a small one. Further, we develop a deep learning-driven mobile application for herb image recognition, which can run entirely on a single smartphone. The application runs in three steps (Fig. 1d). Firstly, image processing is operated on each herb image for the preparation of DNN computation. Then, a DNN is running on the image to get confidence scores for each herb category. Finally, the scores are ranked with top *K* herb categories to display recognition results. The key step is the second one, wherein the proposed network compression algorithm can guarantee the fast response and accurate recognition for good user experience. Experimental results on a public herb image dataset^24^ show that the compressed small DNN can run much faster ($4X{-}5X$) with the near-realtime speed around 20 Frames Per Second (FPS) on common smartphones, while it still maintains a quite comparable recognition accuracy to the original small DNN. Without requiring any hardware, the whole recognition takes place entirely on a common smartphone and it can work completely offline (without internet), which is perfectly suitable for resource-limited settings.

The main contribution of the App in this study is to facilitate the implementation of herb recognition in resources-limited laboratories, hospitals and dispensaries. Firstly, not designed to compete with large DNNs with powerful hardware, they pay more attention to high degree of automation. Secondly, not meant to compete with manual herb recognition, our objective is to alleviate the need of expert resources and chemical materials by being a qualified assistant that can make candidate decisions to accelerate the manual process (Fig. 1). Moreover, we believe this study can promote the accessibility of herbal medicine worldwide and possibly facilitate the collection of valuable herbal medicine data, which has long been a key problem in artificial intelligence and deep learning.

In the following, we explore a deep learning based network compression algorithm for efficient and robust herb image recognition on a single smartphone. Then, we demonstrate that the accuracy of the compressed small DNN is quite consistent to that obtained by the original small DNN. The full procedure is evaluated on a public herb image dataset, and an additional large-scale image classification dataset is also adopted to further validate the effectiveness and generality of the algorithm.

## Related work

In automatic recognition of herbs, researchers first utilize herb images and analyze their low-level appearance for recognition. Chen et al.^9^ construct the color matching template by integrating two observation surfaces of herb pieces, and they show some anti-interference invariance in rotation, shape and color. Liu et al.^10^ extract texture features by converting color images to gray-scale images, wherein the gray level co-occurrence matrix is used for recognition. To distinguish herbs with similar appearance, Ming et al.^11^ combine raman spectroscopy with the support vector machine. However, these studies mainly focus on a few herb categories, due to the fact that low-level property cannot handle large variations in many categories. Recently, inspired by the development of image recognition in computer vision^12–16^ and medical areas^17–23^, deep learning based methods have shown great improvements in herb recognition^24–29^. Sun et al.^24^ apply convolutional neural network in herb image classification, and they also release a herb image dataset with 95 categories. Vo et al.^25^ extract image features by a VGG16-based network^13^, and the features can work well with the LightBGM classifier. To incorporate the expert knowledge, Lai et al.^26^ extract a set of selected traditional features, which are further combined with deep features for joint classification. To deal with the background noise, Zhu et al.^28^ propose the two-way attention method, which combines the instance and category for better discrimination. To assess the feasibility of automated machine learning, Chen et al.^27^ use the open-source platform and built a dataset with 315 commonly used herb categories, but the dataset has not been released yet. To popularize the knowledge of herb medicine, Weng et al.^29^ develop a smartphone application for herb image classification, but the entire process is run on the cloud server. The above studies have used the Deep Neural Network (DNN), which combines with powerful and expensive computation hardware to support the computation. However, due to the high price of the hardware, it is not suited to dispensaries or hospitals in resource-limited settings.

To enable the use of DNN in resource-limited devices, one primary method is to design efficient network structures. One series of studies is the MobileNets. Howard et al.^33^ propose a lightweight DNN, namely MobileNet-V1, for embedded vision applications, and the main contribution is the depth-wise separable convolutions. To further improve the efficiency and accuracy, Sandler et al. design the MobileNet-V2^34^ wherein an inverted residual structure is introduced with removing non-linearities in the narrow layers to maintain representational power. To explore automated search algorithms in network design, Howard et al. further propose the MobileNet-V3^35^, which adopts Neural Architecture Search (NAS) to better tune on the mobile phone CPU. Another series of studies is the ShuffleNets. Zhang et al. introduce the ShuffleNet-V1^36^, which utilizes two new operations, namely pointwise group convolution and channel shuffle, to greatly reduce computation cost while maintaining accuracy. To guide the network design towards direct metrics such as FLOPs, speed and memory access cost, Ma et al. propose the ShuffleNet-V2^37^ to derive several practical guidelines for efficient design. In this paper, we adopt the MobileNet-V2 as the small DNN for its convenient design for subsequent network transfer and cut.

Another primary method is the network compression, which usually contains two main parts, namely network transfer and network cut. In network transfer, Hinton et al.^38^ propose to transfer the logits from the large DNN to the small DNN. To incorporate context information in a single layer or between layers, Zagoruyko et al.^39^ and Yim et al.^40^ construct the attention maps and pairwise relation to improve the accuracy of the small DNN. However, these studies neglected the relation among samples. Park et al. and Liu et al. propose to model the sample relation by the similarity of pairs^41^ and triplets^42^. In this paper, instead of using logits or relation, we adopt the hidden layer for network transfer as it is a compact representation and can be used between different network structures. In network cut, some researchers proposed the sparsity based methods^32,43,44^, but this sparsity is not friendly to hardware acceleration, which requires cutting a whole channel or filter. Based on this fact, the weight based methods have been proposed by jointly cutting network parameters of all the layers with some weight regularization^45–49^, but the accuracy drops obviously due to the difficulty in joint optimization. Motivated by the feature map approximation^50^, we propose a top-down layer-wise network cut method, which is further combined with network transfer to minimize the loss of accuracy in cutting each layer.

## Methods

In this section, we will give a full introduction on how to obtain an efficient and accurate DNN for use on a smartphone. We first give a brief introduction of the DNN and explain the importance of image processing, and then we present a novel deep learning based network compression algorithm to learn a compressed DNN. Finally, we show how to deploy the compressed DNN on the smartphone.

### Brief introduction

**Figure 2:**
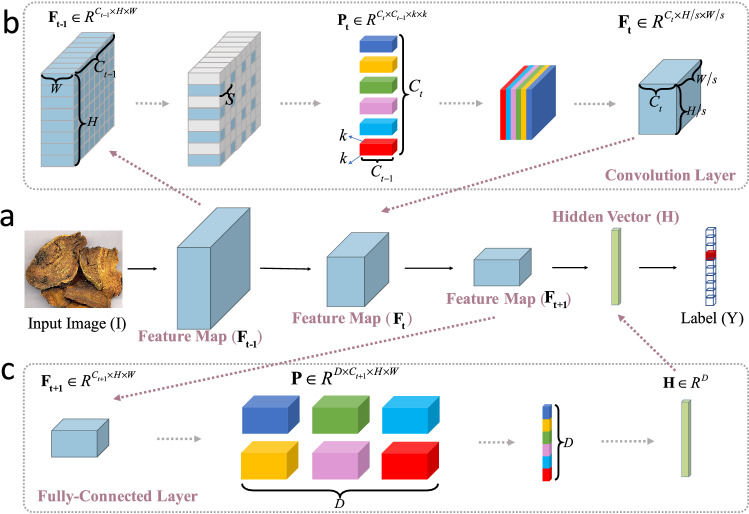
An illustration of a simple DNN for image recognition. (**a**) A simple DNN consists of the feature map, convolution layer, fully-connected layer and hidden vector; (**b**) The convolution layer extracts local information to generate a new map; (**c**) The fully-connected layer aggregates global information to generate the hidden vector.

In this part, we introduce some key concepts and notations in DNN that are related to our method. Figure 2a shows a simple DNN, which contains a sequence of data representation and parameter layers, and it is trained with a one-hot label that denotes which category the herb image belongs to. Among this simple structure, feature map, hidden vector, convolution layer and fully-connection layer are important concepts. The former two are data representation, while the latter two are parameter layers.

#### Feature map

It is a general form of data representation in DNN. It has the form of $\mathbf{{F}} \in {R^{C \times H \times W}}$, wherein *C*, *H* and *W* are the size of the feature map and they denote channel, height and width respectively. For example, an input RGB image has the data size of ${3 \times H \times W}$, wherein the channel *C* is 3. For clarity, we denote the feature map in the ${t}$th layer as ${\mathbf{{F}}_\mathbf{{t}}} \in {R^{{C_t} \times {H_t} \times {W_t}}}$, and we will adopt this notation throughout the paper.

#### Convolution

It can extract information from local regions, and Fig. 2b shows how it works. For the ${t}$th layer, the convolution consists of filters ${\mathbf{{P}}_\mathbf{{t}}} \in {R^{{C_t} \times {C_{t - 1}} \times {k_t} \times {k_t}}}$, wherein ${{C_{t - 1}} \times {k_t} \times {k_t}}$ is the parameter size for a single filter and ${{C_t}}$ is the number of filters. Especially, ${{C_{t - 1}}}$ must equal to the number of channels in the input feature map ${\mathbf{{F}}_{\mathbf{{t - 1}}}}$. Each filter will convolve with local regions in ${\mathbf{{F}}_{\mathbf{{t - 1}}}}$ on every *s* locations to form a new map, and the maps of all ${{C_t}}$ filters are stacked together to generate the output map ${{\mathbf{{F}}}_\mathbf{{t}}} \in {R^{{C_t} \times {H_t} \times {W_t}}}$.

#### Fully-connection

It is a special form of convolution. Given the input feature map ${\mathbf{{F}}_{\mathbf{{t + 1}}}} \in {R^{{C_{t + 1}} \times {H_{t + 1}} \times {W_{t + 1}}}}$, the layer has filters $\mathbf{{P}} \in {R^{D \times {C_{t + 1}} \times {H_{t + 1}} \times {W_{t + 1}}}}$, and these filters act the same to the ones in convolution. But different from the convolution, the layer generates a special form of feature map $\mathbf{{H}} \in {R^D}$, wherein the height and width both equal to 1, as shown in Fig. 2c.

#### Hidden vector

We refer to the output feature map $\mathbf{{H}}$ of the fully-connection layer as the *hidden vector* throughout the paper. The reason we transform the feature map into the hidden vector is to enhance the recognition ability with global image information, and meanwhile prepare the data form for training.

### Image processing

Given a herb image, the objective of image processing is twofolds. Firstly, it processes the image to be prepared for DNN computation. The image size varies a lot due to the different hardware and resolution, but the input image size of the DNN is fixed, e.g. $224 \times 224$ throughout our evaluation. Therefore, it is necessary to process the image to the target size, and this rule applies both in the training and testing phase. Secondly, image processing can be used as an effective data augmentation method to improve the robustness of recognition. We train and evaluate our method on the largest public herb image dataset^24^ that contains 95 herb categories with 5640 herb images, but it is actually a small-scale dataset compared to some large ones in computer vision^51^. Training DNN on this dataset without any data augmentation will easily cause over-fitting, which will degrade the generality of herb image recognition. Particularly, this rule applies mainly at the training phase, while the processing in the testing phase is quite simple.

**Figure 3:**
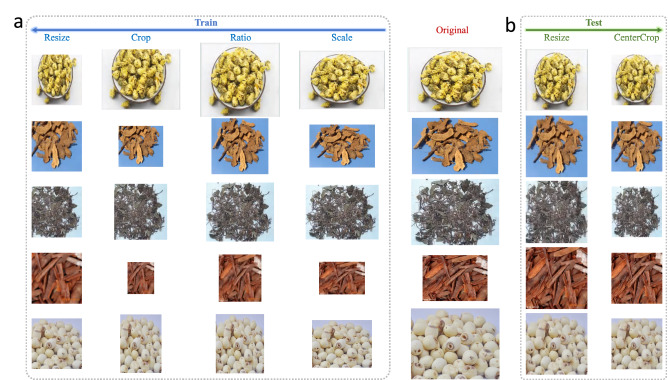
An illustration of data augmentation for training and testing. (**a**) In training, the augmentation includes random scaling, random ratio, random cropping and resizing, and these four steps are processed in order; (**b**) In testing, only resizing and center-cropping are used.

In the training phase, inspired by the data augmentation method in computer vision^15,16^, we use four types of augmentations in order: random scaling, random ratio, random cropping and resizing, as shown in Fig. 3a.

#### Random scaling

The motivation is to adapt to different herb scales caused by the different distance between the herbs and a smartphone. As the smartphone moving away from the herbs, their size in the image will become smaller, which is actually the zoom-out operation. This operation only changes the image scale that is randomly selected, but keeps the width-height ratio fixed, as shown in the “Scale” column in Fig. 3a.

#### Random ratio

The motivation is to adapt to different herb shapes caused by the different angle between the herbs and a smartphone. As the smartphone rotating around the herbs, their shape in the image will be different, i.e. the width-height ratio will change. This operation only modifies the width-height ratio that is randomly selected, but remains the image area unchanged, as shown in the “Ratio” column in Fig. 3a.

#### Random cropping

The motivation is to fully learn the representation ability from every herb detail. Cropping is equivalent to concentrating on a local region, which is actually the zoom-in operation. By this way, DNN has to learn to correctly recognize the herb category from different local regions, which can increase the learning ability from every part of the herbs. Due to the crop location and region are randomly selected, this operation will change both the image scale and width-height ratio, as shown in the “Crop” column in Fig. 3a.

#### Resize

As previously mentioned, the motivation is to prepare for the fixed input size for DNN computation, as shown in the “Resize” column in Fig. 2a.

For each training iteration, the above steps are processed in order to get the input image for DNN computation. With the randomness in each step, the DNN can see various combinations in training, which can largely improve the generality of the herb image recognition. In the testing phase, we only adopt resizing and center-cropping, as used in^15^. The testing image is first resized to $256 \times 256$, then the center region with $224 \times 224$ is cropped to feed the DNN for recognition, as shown in Fig. 3b. Different from the training phase, we want the testing condition to be simple by assuming the herbs are at the near-center region of the image.

### Network compression

In this part, we introduce the deep learning based network compression algorithm, which compresses the large DNN into a small one that can run efficiently and meanwhile maintain considerable accuracy. The algorithm contains three main steps, namely network pre-train, network transfer and network cut. One may wonder why we need an extra cutting step but not designing a small DNN that is small enough? For this question, we will explain it later and validate it in the result part.

#### Network pre-train

Network pre-training is a strategy that trains the DNN initially on a large-scale dataset, and then based on the initialization, the DNN is further trained on the target dataset. The advantage of this strategy is twofolds. Firstly, pre-training is a regularization technique, and it improves recognition generalization ability. Since the DNN is first exposed to a large amount of data, its parameters will be carried to a space that is more likely to represent the data distribution in overall rather than over-fitting a specific subset of underlying data distribution^52,53^. Secondly, pre-training can improve the training stability and speed up the convergence. Deep neural networks, especially those with high representation capacity with tons of parameters, are tend to be vulnerable to random parameter initialization^12^. Pre-training can initialize the parameters in a supervised way, which can provide a good starting point for stable training.

In our strategy, we adopt the ImageNet dataset^51^ as the large-scale set and pre-train the DNN on it, and then the DNN is further trained on the herb image dataset. The ImageNet dataset is a large-scale set in image recognition, and it contains 1000 common object categories and 1.2*M* images^51^. Though its target is not for herb image recognition, it can share general representation, which can bring better recognition generality. Especially, this pre-training rule applies to both the large and small DNN.

**Figure 4:**
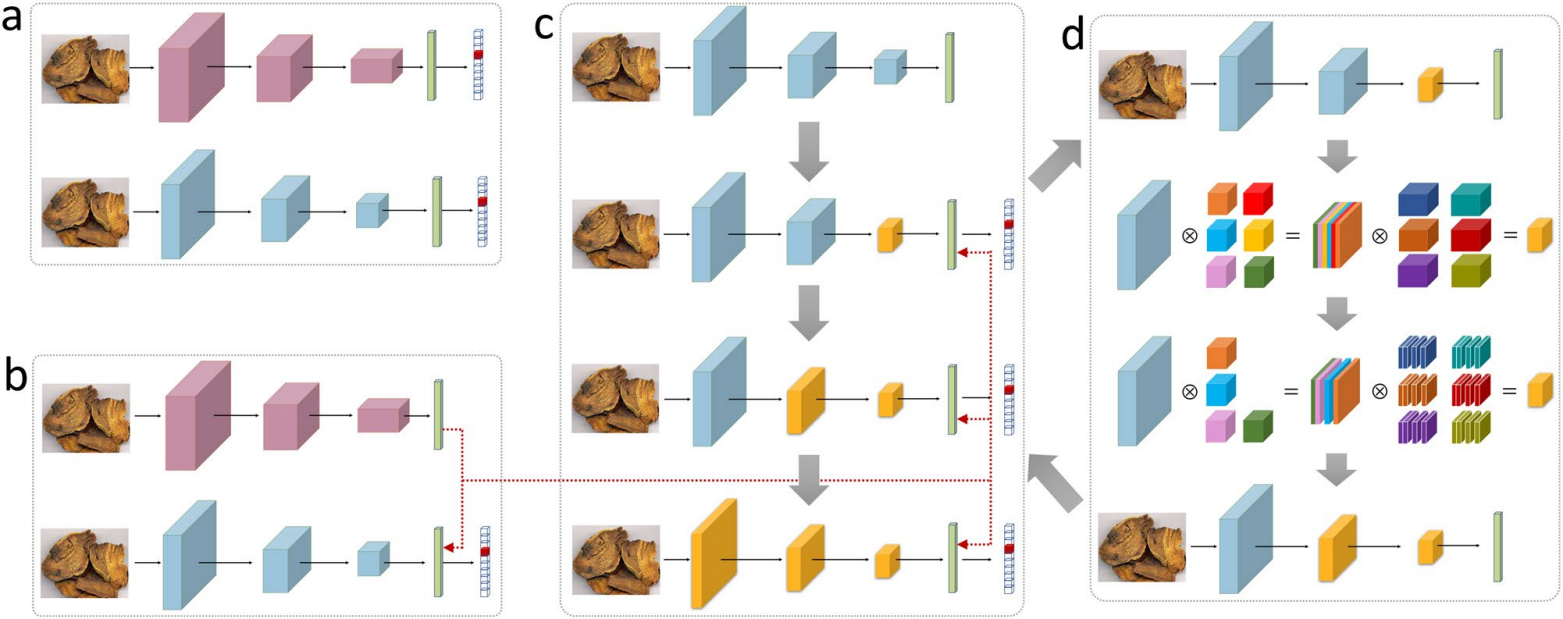
An illustration of the pipeline for the network compression algorithm. (**a**) Firstly, the large and small DNN are pre-trained on the ImageNet dataset and further trained on the herb image dataset, and they are trained individually; (**b**) Secondly, the recognition ability of the large DNN is transferred to the small DNN, as shown by the dotted red line; (**c**) Finally, network cutting is processed in a top-down way from the top layer to the bottom layer; (**d**) The network cutting for a single layer, wherein some unimportant filters are removed.

Let ${\mathbf{{X}}_\mathbf{{i}}} \in {R^{3 \times H \times W}} \;\; (i = 1,...,N)$ be the input RGB image, wherein *H* and *W* are the height and width, and *N* is the number of herb images. For each image ${\mathbf{{X}}_\mathbf{{i}}}$, the recognition label is defined as ${\mathbf{{Y}}_\mathbf{{i}}} \in {R^C}$, which is a one-hot vector with *C* herb categories. We denote the pre-trained large and small DNN as ${W_L}$ and ${W_S}$ respectively, and they are further trained on the herb image dataset with the cross-entropy loss in a mini-batch way:

1 $$\begin{aligned} \begin{array}{l} L({W_L}) = \min {{\sum \nolimits _{\mathrm{{i}} = 1}^B { - {\mathbf{{Y}}_\mathbf{{i}}}\log \phi ({\mathbf{{X}}_\mathbf{{i}}})} } \big / B},\\ L({W_S}) = \min {{\sum \nolimits _{\mathrm{{i}} = 1}^B { - {\mathbf{{Y}}_\mathbf{{i}}}\log \varphi ({\mathbf{{X}}_\mathbf{{i}}})} } \big / B} \end{array} \end{aligned}$$

wherein ${\phi ({\mathbf{{X}}_\mathbf{{i}}})}$ and ${\varphi ({\mathbf{{X}}_\mathbf{{i}}})}$ are the softmax output of the large and small DNN respectively, and *B* is the training batchsize. In this step, both DNNs are trained individually, i.e. without any interaction between them, as shown in Fig. 4a. With enough training, they can learn some recognition ability for herb image recognition.

#### Network transfer

After the pre-training, the large DNN is fixed and all the subsequent steps will be operated only on the small DNN. Due to the much fewer parameters, the small DNN cannot learn the recognition ability as good as the large one, thus its recognition accuracy is usually lower. To reduce the accuracy gap, we propose to transfer the recognition ability from the large DNN to the small one.

Some studies in computer vision have found that given the same input image, if the feature map of the small DNN can be close to the one of the large DNN, then their predicted category will also be similar, thus their recognition ability can be comparable^39–42,54,55^. Based on this understanding, we can formulate the transfer process to be matching the feature map of both networks in a specific layer. As each network has dozens of layers, one problem is which layer to use. Inspired by the fact that the layer of higher level has better semantic representation^38,52,53,55^, we propose to use the hidden vector $\mathbf{{H}}$, as it is a compact and high-level representation. Let ${\mathbf{{H}}_\mathbf{{L}}} \in {R^D}$ and ${\mathbf{{H}}_\mathbf{{S}}} \in {R^D}$ be the hidden vector for the large and small DNN respectively, then the network transfer on the small DNN can be formulated with the additional transfer loss as follows:

2 $$\begin{aligned} L({W_S}^K\left| {{W_S}} \right. ) = \min {{\sum \nolimits _{i = 1}^B {( - {\mathbf{{Y}}_\mathbf{{i}}}\log \varphi ({\mathbf{{X}}_\mathbf{{i}}}) + \lambda \left\| {{\mathbf{{H}}_\mathbf{{S}}} - {\mathbf{{H}}_\mathbf{{L}}}} \right\| _2^2)} } \big / B}, \end{aligned}$$

wherein the transferred small DNN is denoted as ${W_S}^K$, and it is trained based on the previously trained ${{W_S}}$. Besides, $\lambda$ is the weight of the network transfer that controls how much ability should be transferred from the large DNN. In this way, the recognition ability can be transferred to the small DNN, which maintains the high accuracy of the large DNN but runs faster at the same time, as shown in Fig. 4b.

#### Network cut

The objective of this step is to improve the network efficiency by cutting its parameters, which can reduce the amount of computation. As mentioned before, one confusing idea is why we need an extra cutting step but not using a small DNN that is small enough? Actually, in network transfer, the gap in the parameter number of the large and small DNN cannot be too large. As the small DNN only has limited learning ability, the large gap cannot guarantee the efficacy of the transfer, and this observation will be validated in the result part. This implies the small DNN cannot be too small, but it causes another problem that the small DNN cannot achieve high efficiency at the same time. Therefore, we propose a network cutting method to achieve the best trade-off between accuracy and efficiency.

Before going into it, we need to ask three questions: (1) which parameters should be cut? (2) what if the accuracy drops after cutting them? (3) do we cut all layers jointly or each layer individually? In fact, the answers constitute the three steps in network cutting. Inspired by some recent studies^47,48,56^, we will give our answers for these questions as follows.

For the ${t}$th layer, cutting parameters is equivalent to removing unimportant filters in ${\mathbf{{P}}_\mathbf{{t}}} \in {R^{{C_t} \times {C_{t - 1}} \times k \times k}}$, i.e. to reduce the filter number ${{C_t}}$. To measure the importance of each filter, one intuitive idea is to see how much it affects subsequent feature maps if we remove it, and the unimportant ones will have lower influences. Specifically, assume one filter has been removed, then we will have ${\mathbf{{P}}_\mathbf{{t}}} \in {R^{({C_t} - 1) \times {C_{t - 1}} \times k \times k}}$, ${\mathbf{{F}}_\mathbf{{t}}} \in {R^{({C_t} - 1) \times H \times W}}$, ${\mathbf{{P}}_{\mathbf{{t + 1}}}} \in {R^{{C_{t + 1}} \times ({C_t} - 1) \times k \times k}}$ and ${\mathbf{{F}}_{\mathbf{{t + 1}}}} \in {R^{{C_{t + 1}} \times H \times W}}$. We see that ${\mathbf{{F}}_{\mathbf{{t + 1}}}}$ is the nearest feature map with its size unchanged, thus we use it as the reference map. To measure the importance, we use a subset of herb images and calculate the reconstruction error of the reference map by removing each filter individually:

3 $$\begin{aligned} Scor{e^{{C_i}}} = {{\sum \nolimits _{m = 1}^M {\left\| {\mathbf{{F}}_{\mathbf{{t + 1}}}^{{C_i}}({\mathbf{{X}}_\mathbf{{m}}}) - {\mathbf{{F}}_{\mathbf{{t + 1}}}}({\mathbf{{X}}_\mathbf{{m}}})} \right\| _2^2} } \big / M}, \end{aligned}$$

wherein $\left\{ {{\mathbf{{X}}_\mathbf{{m}}}|m = 1,...,M,M \ll N} \right\}$ is the sampled subset, ${\mathbf{{F}}_{\mathbf{{t + 1}}}^{{C_i}}({\mathbf{{X}}_\mathbf{{m}}})}$ is the feature map for image ${{\mathbf{{X}}_\mathbf{{m}}}}$ by removing the ${i}$th filter from ${\mathbf{{P}}_\mathbf{{t}}}$, and $Scor{e^{{C_i}}}$ is the importance score by removing the ${i}$th filter from ${\mathbf{{P}}_\mathbf{{t}}}$. By ranking the importance scores, we can cut the filters with smaller scores by a given cutting ratio $\alpha$, i.e. $\left\lceil {{C_t} \times \alpha } \right\rceil$ filters will be cut and $\left\lceil {{C_t} \times (1 - \alpha )} \right\rceil$ filters will be reserved, which gives the new filters ${\mathbf{{P}}_\mathbf{{t}}} \in {R^{\left\lceil {{C_t} \times (1 - \alpha )} \right\rceil \times {C_{t - 1}} \times k \times k}}$ for the ${t}$th layer, as shown in Fig. 4d.

The unimportant filters, though they contribute less to the recognition, they still learn some image representation that can be helpful. Therefore, simply removing them will cause the loss of recognition ability^43,57^. To recover the loss back, one common solution is to re-train the after-cut network^56,58^, and this can make the reserved filters fully learn the image presentation that is necessary for the recognition. Specifically, we use all the herb images to re-train the after-cut small DNN, and the same training rule in Eq. (2) is adopted:

4 $$\begin{aligned} L({W_S}^t{\left| {{W_S}} \right. ^K}) = \min {{\sum \nolimits _{i = 1}^B {( - {\mathbf{{Y}}_\mathbf{{i}}}\log \varphi ({\mathbf{{X}}_\mathbf{{i}}}) + \lambda \left\| {{\mathbf{{H}}_\mathbf{{S}}} - {\mathbf{{H}}_\mathbf{{L}}}} \right\| _2^2)} } \big / B}, \end{aligned}$$

wherein ${W_S}^t$ is the re-trained small DNN for the ${t}$th layer, and it is trained based on the transferred small DNN ${W_S}^K$ with cutting the filters at the ${t}$th layer. With the re-training step, the accuracy can be well recovered, and the whole cutting process at the ${t}$th layer is then finished, as shown in Fig. 4d.

By far, we have answered the first two questions. For the third one about cutting all layers jointly or each layer individually, we adopt an iterative approach that cuts layers one by one. Assume there are *T* filter layers in the transferred small DNN ${W_S}^K$, and we start the cutting from layer *T* all the way down to layer 1. For each layer *t*, the cutting is very similar to the above two steps with only one difference in Eq. (4): the top layer *T* ($t = T$) is trained based on the after-cut ${W_S}^{K}$, while others ($1 \le t < T$) use the after-cut ${W_S}^{t+1}$ as initialization, as shown in Fig. 4c. Especially, the cutting ratio $\alpha$ can be adjusted to satisfy different efficiency requirements, but we should also pay attention to the trade-off between accuracy and efficiency. After the top-down iterative cutting on each layer, ${W_S}^{1}$ is the final compressed small DNN for deployment on smartphones.

#### A short summary

To give a short summary of the network compression pipeline, we conclude the algorithm pipeline as follows. 
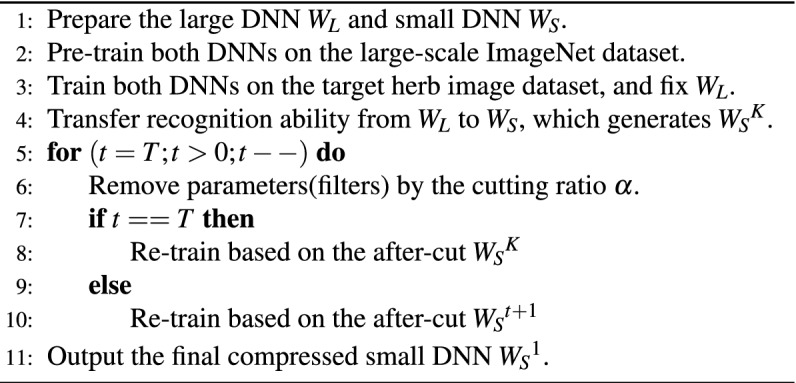


### Network deployment

After the network compression, we deploy the final compressed small DNN on a smartphone. Given a smartphone, a video sequence is first taken from the camera, and the sequence is split into each individual image frame. Then, the data augmentation for testing will be operated in each frame to generate the input data, as shown in Fig. 3b. Finally, the compressed small DNN will run on each input data to output the ranked predicted herb categories. All these steps are illustrated in Fig. 1c.

## Results

We first introduce experimental settings, then the efficacy of image processing and network compression will be validated, lastly we show the running efficiency on three common smartphones.

### Experimental setting

The setup is given based on seven parts: dataset, image processing, network selection, network pre-train, network transfer/cut, network deployment and evaluation.

#### Dataset

We use the public largest herb image dataset released in^24^. It contains 95 herb categories with 5640 images, which are all collected from the internet with the herbs having mutual occlusion and cluttered backgrounds. Some herb images in the dataset are shown in Fig. 3. For each herb category, the number of herb images varies from 15 to 180, and we randomly split them into $70\%$ training and $30\%$ testing, which contain 3904 and 1736 images respectively. This random split is repeated 5 times for fivefold cross-validation.

#### Image processing

In training, we use a four-step image processing method for each image. (1) Random Scale: the raw image is scaled with the scale randomly selected in $\left[ {0.08,\,1.0} \right]$; (2) Random Ratio: the scaled image is reshaped to different width-height ratios with the ratio randomly selected in $\left[ {3/4,\,4/3} \right]$; (3) Random Crop: the ratio image is cropped with the crop location and region size randomly selected within the image; (4) Resize: the cropped image is resized to $224 \times 224$. In testing, we use a two-step image processing method for each image. (1) Resize: the original image is resized to $256 \times 256$; (2) Center Crop: the center region with $224 \times 224$ is cropped. For both training and testing, the result image is converted into the Float type ([0, 1]) and normalized with mean and variance to generate the input data for DNN computation.

#### Network selection

We adopt the standard ResNet-50^15^ and MobileNet-V2^34^ as the large and small DNN respectively. The two DNNs have different network structures, wherein the large one uses deep residual blocks for better accuracy^15^, while the small one uses separable depth-wise convolutions and inverse bottleneck for fewer parameters and higher efficiency^34^.

#### Network pre-train

For the pre-training on the ImageNet^51^ dataset, both DNNs start with the learning rate of 0.1 and ends with 0.0001, and each rate is divided by 10 for every 30 epochs, i.e. four rates with 120 epochs are used in total. For the training on the herb image dataset, the large and small DNN are trained with the starting rate of 0.01 and the ending rate of 0.0001, and each rate is divided by 10 for every 5000/10,000 iterations, i.e. three rates with 15,000/30,000 iterations are used in total. Besides, the optimizer of stochastic gradient descent is adopted with the batchsize of 32, momentum of 0.9, and weight decay of 0.0005 throughout the whole evaluation.

#### Network transfer/cut

In network transfer, the training starts with the rate of 0.001 and ends with 0.0001, and the number of iterations for each rate is set to be 10,000 and 5000 respectively. Besides, the transfer weight $\lambda$ in Eq. (2) is set to be 1.0. In network cut, we use the same learning rates as in the transfer process, while the number of iterations for each rate is set to be 5000 and 2000. Especially, we use the cutting ratio of 0.5 for the best trade-off between accuracy and efficiency, which will be validated later.

#### Network deployment

We train and evaluate our DNNs with the open-source PyTorch framework^59^, which also provides the tool to deploy the DNN on smartphones. We deploy the final compressed DNN based on the Android system, and three smartphones are used with their increasing CPU computation ability: HUAWEI Mate9 Pro, Xiaomi MI 9 and HUAWEI P30 Pro. For use in low-cost situations, the smartphones we have used are rather common and typical, as all of them have been released for more than two years with a low price.

#### Evaluation

The proposed method will be evaluated based on accuracy and efficiency. For the accuracy part, it is computed by dividing the total number of images with the number of correctly recognized images in each category, and we report the $\text {top}1$ and $\text {top}5$ average accuracy of all 95 categories. Especially, due to the training and testing sets have been randomly selected with 5 times for the fivefold cross-validation, the mean and variance of the average accuracy will also be reported. For the efficiency part, we evaluate the time cost of image processing and DNN computation, and the average time cost with 100 runs on the smartphone CPU will be reported.

### Image processing

In this part, we validate the effectiveness of the training data augmentation. We adopt the small DNN for evaluation, and the top1/top5 average accuracy will be reported, wherein topn denotes if the correct category is in the top n predicted categories. Table 1a shows the results of the small DNN with and without image processing. We see that training without processing obtains the top1/top5 accuracy of $64.65\%/86.02\%$ respectively, while the extra processing step gives about $4\%/2\%$ improvement to $68.89\%/88.03\%$. These improvements imply the image processing can be effective in enhancing the robustness and generality of the herb image recognition, and we will give more insights to explain the improvements.

**Table 1:** A summary of main results of the image processing and network compression.

DNN strategy	Top1 Mean ± std (%)	Top5 Mean ± std (%)
**a. Image processing**		
Small DNN w/o image processing	64.65 ± 1.09	86.02 ± 0.51
Small DNN + image processing	68.89 ± 0.69	88.03 ± 0.69
**b. Network pre-train**		
Small DNN w/o pre-train	46.51 ± 0.90	75.38 ± 1.48
Small DNN + pre-train	68.89 ± 0.69	88.03 ± 0.69
**c. Network transfer**		
Large DNN	70.61 ± 1.00	88.97 ± 0.41
Small DNN	68.89 ± 0.69	88.03 ± 0.69
Small DNN + transfer	70.97 ± 0.50	88.72 ± 0.79
Small DNN w/o pre-train + transfer	55.55 ± 3.22	79.25 ± 2.11
**d. Network cut**		
Small DNN + transfer	70.97 ± 0.50	88.72 ± 0.79
Small DNN + cut($\alpha =0.5$, $\lambda =0$)	67.62 ± 0.84	85.69 ± 0.85
Small DNN + cut ($\alpha =0.5$, $\lambda =1$)	69.44 ± 1.10	87.66 ± 0.73
Small DNN + transfer + cut ($\alpha =0.5$, $\lambda =0$)	68.01 ± 0.47	85.88 ± 0.54
Small DNN + transfer + cut ($\alpha =0.5$, $\lambda =1$)	69.98 ± 1.09	87.71 ± 0.73
**e. Network structure**		
Small DNN + transfer + cut ($\alpha =0.5$, $\lambda =1$)	69.98 ± 1.09	87.71 ± 0.73
New small DNN	62.66 ± 1.19	84.91 ± 1.06
New small DNN + transfer ($\lambda =1$)	66.68 ± 1.21	86.44 ± 0.78

**Figure 5:**
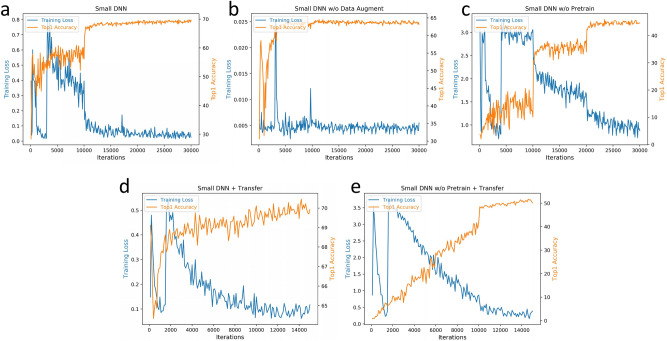
The training loss and top1 accuracy on different networks. (**a**) Small DNN with the data augmentation and network pre-train; (**b**) Small DNN without the data augment; (**c**) Small DNN without the network pre-train; (**d**) Small DNN using the network transfer; (**e**) Small DNN without the pre-train but using the network transfer.

Figure 5a,b show the change of training loss and top1 accuracy along the training process with and without the augmentation, and there are two main observations. Firstly, training without augmentation (Fig. 5b) converges quickly within 5000 iterations and achieves the training loss of 0.005, which is 10 times lower than the one with augmentation (Fig. 5a) that converges slowly with 20,000 iterations. Secondly, the top1 accuracy without augmentation quickly saturates around 10,000 iterations but then slightly decreases, while the one with augmentation keeps going up to a higher level with the increasing number of iterations. When the augmentation is adopted, different training samples are generated in each iteration, and the enough training can fully learn the representation from every herb detail and also simulate the different setup between the camera and herbs, which can easily adapt to different testing situations with high robustness and generality. However, if the augmentation is removed, the training will always see the same samples, and the limited number of training samples cannot learn the herb detail and generalize to different testing situations, which causes the over-fitting to happen. These observations indicate that data augmentation can effectively overcome the over-fitting problem by learning better recognition generality.

**Figure 6:**
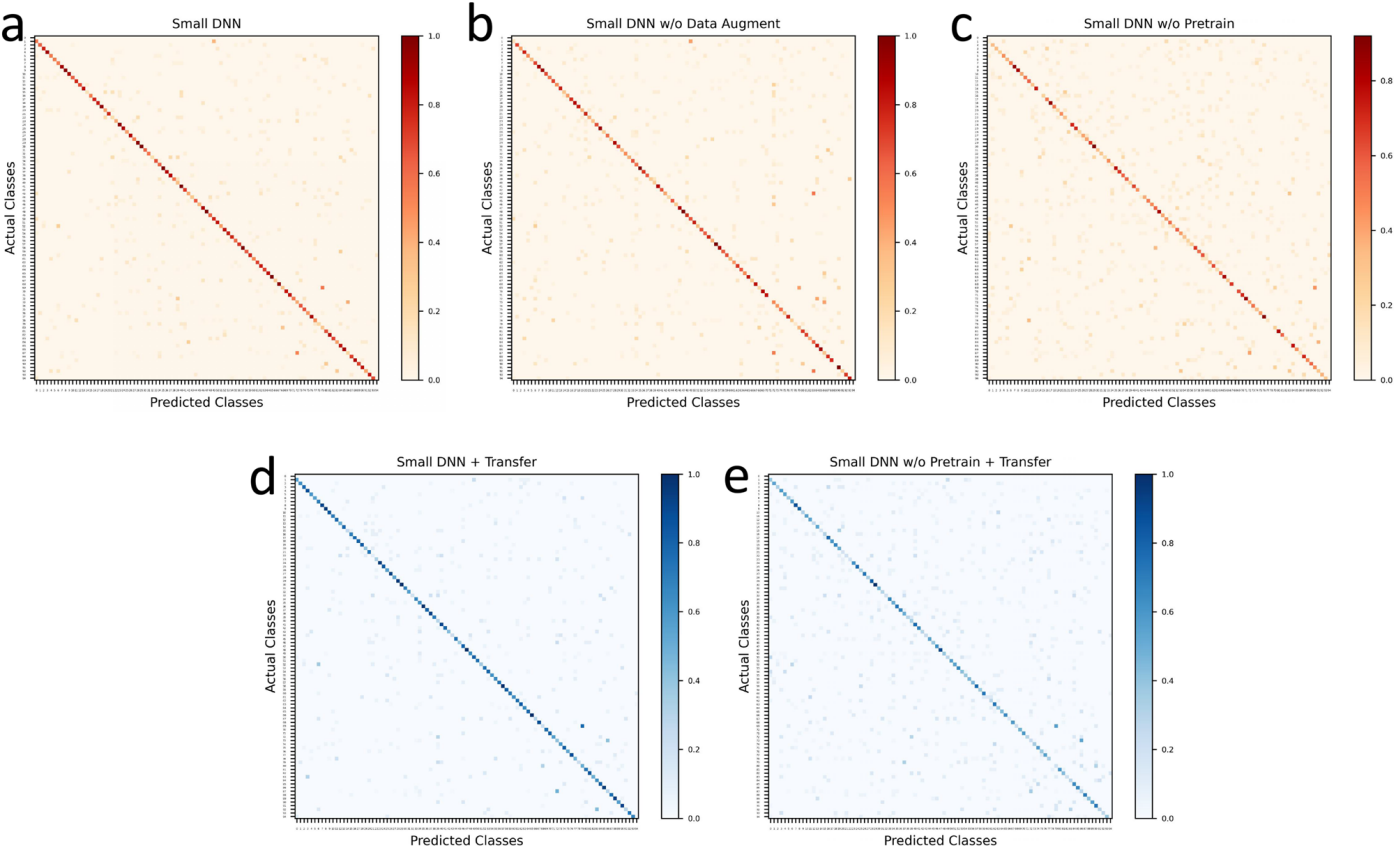
The confusion matrix of different networks on the testing set. (**a**) Small DNN with the data augmentation and network pre-train; (**b**) Small DNN without the data augment; (**c**) Small DNN without the network pre-train; (**d**) Small DNN using the network transfer; (**e**) Small DNN without the pre-train but using the network transfer.

Figure 6a,b give a visualization of the recognition ability by comparing the confusion matrix with and without data augmentation. One main observation is that in the matrix without augmentation (Fig. 6b), there are more red points than the one with augmentation, especially in the right area, and these points indicate that the corresponding true herb categories are easily confused by others. We further realize that for most of these confused herb categories, there are not enough training samples, as the number of training samples ranges from 10 to 90 across the dataset. Without augmentation, the limited number of training samples cannot well learn the discriminability between the true category and others, which results in the confusion. By using the data augmentation as in Fig. 6a, more training samples can be generated to incorporate the sample diversity, which can alleviate the confusion problem in some degree.

### Network pre-train

In this part, we validate the effectiveness of network pre-train in the network compression pipeline. Similar to the setup in image processing, the small DNN is adopted for evaluation and the top1/top5 average accuracy will be reported. Table 1b shows the results of the small DNN with and without the network pre-train. It can be clearly observed that training without the pre-train only gets the top1/top5 accuracy of $46.51\%/75.38\%$, while the small DNN with pre-train has achieved $68.89\%/88.03\%$, which has obtained a huge improvement of $22\%/13\%$ respectively. This impressive increase implies that the network pre-train has played a vital role in training the DNN. As mentioned in the method part, the pre-train not only brings better recognition generality, but also improves training stability and speeds up the convergence with good initialization. We will give more insights based on these two points.

Figure5a,c show the change of training loss and top1 accuracy along the training process with and without the network pre-train, and we have two main observations. Firstly, the small DNN without pre-train (Fig. 5c) only gets the ending loss around 1.0, which is much higher than the one with pre-train (Fig. 5a) that is around 0.05. Secondly, the loss without the pre-train decreases slowly within 30,000 iterations, while the small DNN with pre-train converges quickly around 10,000 iterations. When training a network, shallow layers can learn low-level representation such as lines, circles and squares^60^. As going deeper into the network, we can learn mid-level representation such as intricate patterns or combinations of low-level shapes. And finally, the deepest layers of the network can learn high-level representation that is specific for target object categories. With pre-training on a large-scale dataset such as ImageNet^51^, the large number of categories and images can make the network learn rich image representation of different levels, wherein the low-level and mid-level representation can be general and transferred across different tasks^60^. Due to this representation generality, the network can quickly adapt to the new task and re-learn only the high-level part efficiently. Combined with the stable initialization given by the supervised pre-training process, the pre-train can largely improve the training stability and speed up the convergence.

Figure6a,c also visualize the recognition ability by showing the confusion matrix of the small DNN with and without the network pre-train. It can be observed that there are many light-colored points in the matrix without the pre-train (Fig. 6c), especially at the upper-right region. Given the fact that the top1 accuracy without pre-train is only $46.51\%$, this observation indicates many categories are easily confused by others, which also explains that the color of the diagonal points is not as dark as the one with pre-train. Without the pre-training, due to the limited number of the categories and images in the small-scale herb image dataset, the representation of different levels cannot be fully learned, which will cause the large drop of the recognition ability and accuracy.

### Network transfer

In this part, we validate the effectiveness of the network transfer in the network compression pipeline. Table 1c shows the top1/top5 accuracy of the large DNN, small DNN and the small DNN with network transfer. The small DNN obtains the top1/top5 accuracy of $68.89\%/88.03\%$, which is a little lower than the large DNN with $70.61\%/88.97\%$, and this small gap is caused by the different learning capacity that is determined by the number of network parameters. However, by using the transfer, the small DNN can surpass the large DNN and achieve the top1 accuracy of $70.97\%$, which is very encouraging as the small DNN can beat the large DNN with a much smaller number of parameters. We further evaluate the influence of using no pre-train in the transfer process, and there is a large drop on the top1/top5 accuracy, which only gets $55.55\%/79.25\%$ respectively. This implies that the pre-train cannot be simply replaced by network transfer. We will provide some insights into these results.

The result of the transferred small DNN beating the large one implies two facts. For the first fact, the large DNN may suffer from the over-fitting problem. Due to the large gap in the parameter number between the large DNN and the small one, we can make the small DNN approximate the large one, but hard to surpass it. Besides, it is unlikely that the large DNN has not been well trained with data augmentation, network pre-train and enough iterations, thus the most probable explanation is the over-fitting. Though pre-training on the ImageNet dataset can largely alleviate the problem, the difference on the amount of training data between the ImageNet and our task is quite huge (categories: 1000 vs 95, images: 1.2*M* vs 5.4*k*), thus the large DNN will suffer from some over-fitting. For the second fact, the small DNN without the network transfer may be optimized to a local minimum. As the DNN is usually trained with a large number of training samples, it can only be optimized with stochastic optimization methods in a mini-batch way, but the method such as Stochastic Gradient Decent (SGD) generally converges to the local minimum. However, when transferring the stronger recognition ability from the large DNN, the transfer can be used as an auxiliary task to help the optimization go to a better direction to the global minimum. Combined with the over-fitting of the large DNN, the transferred small DNN may beat the large one.

Given the fact that the large DNN has been pre-trained, one may wonder if we transfer the recognition ability from the large DNN, is it necessary to pre-train the small DNN? The answer is still yes, and Fig. 5d,e give the comparison on the training loss and top1 accuracy. It can be clearly observed that without the network pre-train (Fig. 5e), the small DNN gets a much higher ending loss and a much lower accuracy, which indicates that the transfer step cannot make the small DNN learn the same recognition ability as the pre-train does. The main reason comes from the fact deep neural networks are tend to be vulnerable to random parameter initialization. Without the good starting point given by the supervised pre-training, it is difficult to learn the recognition ability effectively and efficiently. Figure 6d,e also visualize this observation, and basically we observe the same phenomenon happens as in the pre-train part.

### Network cut

In this part, we validate the effectiveness of the network cut, which will be evaluated from three aspects. Firstly, the cutting ratio $\alpha$ will be selected to achieve the best trade-off between accuracy and efficiency. Then, we will show the benefits of the network transfer in recovering the loss of recognition ability. Finally, we show the cutting step is important to obtain a powerful small DNN. Particularly, the ratio $\alpha$ is first evaluated in one split of the training and testing data, and then the best $\alpha$ is used for fivefold cross-validation in the latter two aspects.

**Figure 7:**
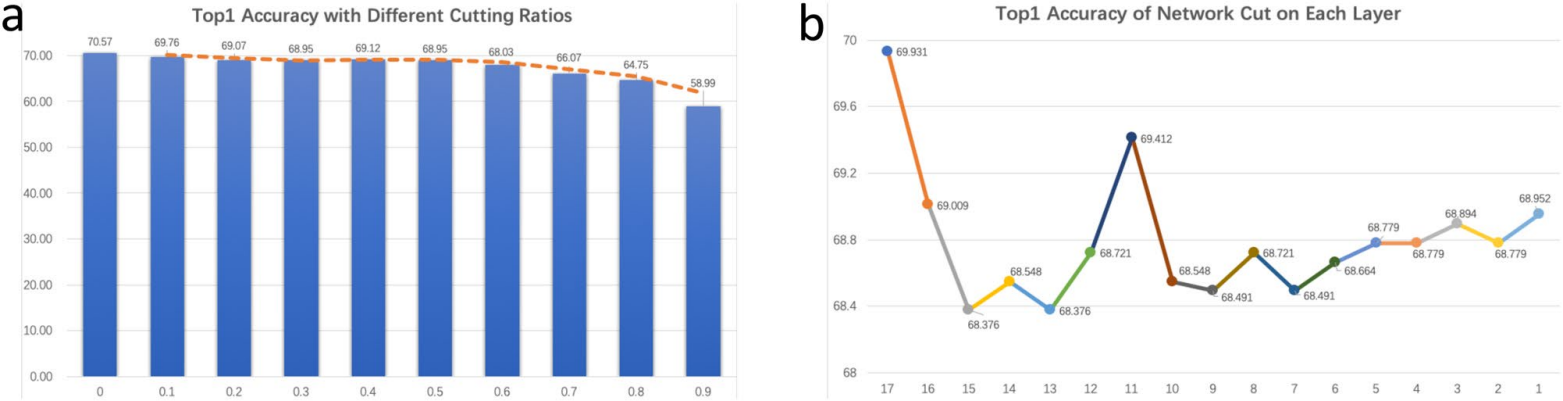
The evaluation of the network cut on different cutting ratios and different cutting layers. (**a**) The top1 accuracy on different cutting ratios; (**b**) The top1 accuracy on different cutting layers with the fixed ratio of 0.5.

Figure 7a shows the top1 accuracy of the small DNN with the cutting ratio increasing from 0.1 to 0.9. It can be observed that the accuracy drops a little by $0.8\%$ and $1.5\%$ at the ratio of 0.1 and 0.2 respectively, and it remains basically stable with the ratio increasing from 0.2 to 0.5, while the accuracy begins to drop after the ratio of 0.5. Consider the trade-off between accuracy and efficiency, we select $\alpha =0.5$ as the best ratio, which indicates that the small DNN is just half the size but with only a minor loss on accuracy. Figure 7b shows the change of top1 accuracy with the selected ratio of 0.5 applied on each layer in the small DNN, which contains 17 layer blocks and will be cut in a top-down way from layer 17 to layer 1. We observe that there are some drops on the accuracy at the top layers such as 17/16/11, but with the network going down to the shallow layers, the accuracy gradually increases to the level that can be comparable to layer 16. One main reason is the top layers learn high-level representation that can be highly related to target categories, and cutting them will harm the recognition ability. While for the shallow layers, they learn more general and common low-level and mid-level representation that transferred from the pre-trained task, and removing some unimportant representation can make the shallow layers focus more on the target task. We use $\alpha =0.5$ throughout our evaluation to make the best trade-off between accuracy and efficiency.

Table 1d shows the influence of the network transfer and the after-cut re-training with transfer loss on the top1/top5 accuracy, wherein $\alpha$ is the cutting ratio and $\lambda$ is the transfer weight in Eq. (4). We have three main observations. Firstly, by using no network transfer as initialization (*small DNN + cut*), the cutting process with the re-training transfer loss ($\lambda =1$ in Eq. (4)) achieves the top1/top5 accuracy of $69.44\%/67.62\%$, which is about $1.8\%/2.0\%$ higher than the one without the transfer ($\lambda =0$ in Eq. (4)). This indicates that the transfer can effectively recover the loss of recognition ability while cutting unimportant parameters. Secondly, by adopting the network transfer as initialization (*small DNN + transfer + cut*), the top1/top5 accuracy is stably higher than the one without the initialization (*small DNN + cut*), e.g. the improvement of top1 accuracy is about $0.5\%$ for both setup ($\lambda =0/\lambda =1$). Given the fact that the transferred small DNN has improved the top1/top5 accuracy by $1.9\%/0/7\%$ over the small DNN (Table 1c), it can provide better initialization to stabilize the cutting process. Finally, we see that the final compressed small DNN is very competitive (*small DNN + transfer + cut* ($\alpha =0.5$, $\lambda =1$)). Compared to the small DNN, we can still obtain $1\%$ improvement on the top1 accuracy, but only with half the network size. Meanwhile, compared to the large DNN, though there is a minor loss about $0.6\%$ on the top1 accuracy, but we can achieve much higher efficiency with much less computation cost, which will be shown later.

Finally, we answer the question about instead of using the extra cutting step, why not using a small DNN that is small enough? To validate the importance of the network cut, we adopt the same network structure of the final compressed small DNN to construct a new network, which we call the new DNN. Based on the compressed pipeline, we make the new DNN go through the network pre-train and network transfer, and then its top1/top5 accuracy is compared with the final compressed small DNN, as shown in Table 1e. It can be observed that the new DNN only achieves the top1/top5 accuracy of $62.66\%/84.91\%$, which is much lower than the small DNN with $68.89\%/88.03\%$, and this is reasonable as the new DNN has only half the parameters and has weaker recognition ability. However, when using the network transfer, the accuracy of the new DNN is still $3.3\%/1.3\%$ lower than the final compressed small DNN, which implies the network cut is important in preserving the recognition ability while cutting the parameters. Actually, in network transfer, the gap in the parameter number of the large and small DNN cannot be too large. As the new DNN only has limited learning ability, the large gap cannot guarantee the efficacy of the network transfer.

### Network deployment

In this part, we evaluate the running efficiency of the final compressed DNN on smartphones. As we develop the app with android, three android phones are selected in the evaluation: HUAWEI Mate9 Pro, Xiaomi MI 9 and HUAWEI P30 Pro. Table 2 shows their detailed configuration based on CPU type, basic frequency, RAM size, android version and screen resolution. There are two main reasons why we select them. Firstly, they are common and typical, and all have been released for more than two years with a low price, which can be widely accepted by resource-limited dispensaries or hospitals. Secondly, they have different computation ability ranging from the low-level HUAWEI Mate9 Pro to the mid-level HUAWEI P30 Pro, where we can evaluate the efficacy and efficiency of the compression algorithm on different low-cost situations.

**Table 2:** The efficiency evaluation on three common smartphones with detailed configuration.

Smartphone		HUAWEI Mate9 Pro	Xiaomi MI 9	HUAWEI P30 Pro
CPU type		Kirin 960	Qualcomm 855	Kirin 980 + NPU
Basic frequency		2.36 GHz	2.83 GHz	2.60 GHZ
RAM size		4 GB	8 GB	8 GB
Android version		9	9	10
Screen resolution		1080 x 1920	1080 x 2340	1080 x 2340
Large DNN	Average running time	342 ms	255 ms	187 ms
	Frames per second	2.9 fps	3.9 fps	5.3 fps
	Top1 accuracy	70.61 ± 1.00		
	Top5 accuracy	88.97 ± 0.41		
Small DNN + transfer	Average running time	130 ms	73 ms	59 ms
	Frames per second	7.7 fps	13.6 fps	16.7 fps
	Top1 accuracy	70.97 ± 0.50		
	Top5 accuracy	88.72 ± 0.79		
Small DNN + transfer + cut	Average running time	75 ms	51 ms	48 ms
	Frames per second	13.4 fps	19.6 fps	21.0 fps
	Top1 accuracy	69.98 ± 1.09		
	Top5 accuracy	87.71 ± 0.73		

**Figure 8:**
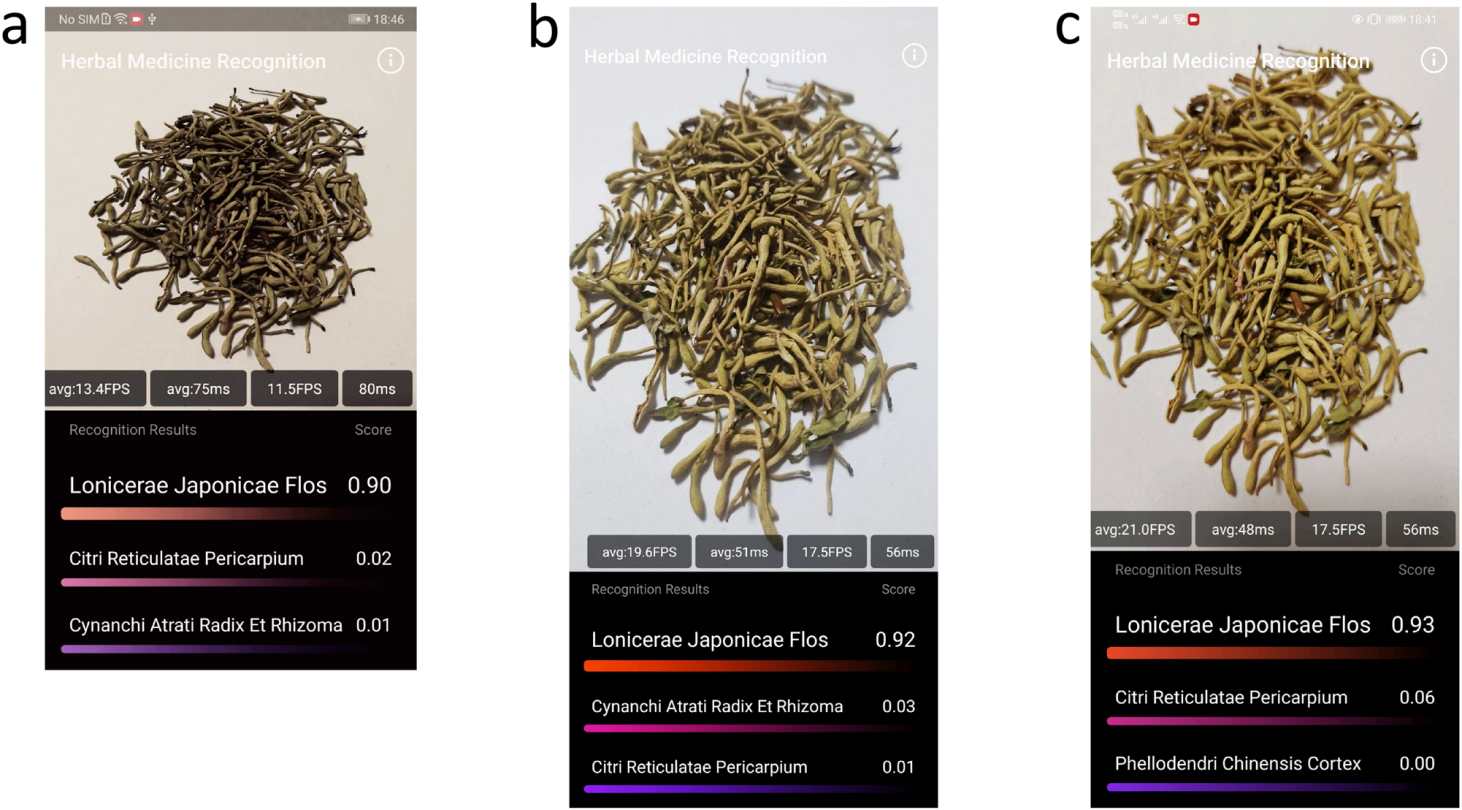
The screenshots of the application interface on three smartphones with their true resolution ratio. (**a**) HUAWEI Mate9 Pro; (**b**) Xiaomi MI 9; (**c**) HUAWEI P30 Pro.

Table 2 shows the average running time (ms) and frames per second (fps) of the large DNN, the transferred small DNN (*small DNN + transfer*) and the final compressed small DNN (*small DNN + transfer + cut*) on the three smartphones, and we have two main observations. Firstly, from the viewpoint of different networks, the transferred small DNN can achieve the speedup of $2.6X{-}3.5X$ over the large DNN, and it also obtains a higher top1 accuracy. Meanwhile, the final compressed small DNN can further improve by $1.2X{-}1.7X$ over the transferred small DNN. Thus the final DNN has achieved the total speedup of $4X{-}5X$ over the large DNN, but with only a minor loss on the top1 accuracy, which demonstrates the network compression can largely reduce the computation cost for use in low-cost situations. Secondly, from the viewpoint of different devices, Mate9/Mi9/Mate30 have achieved the final frequency of $13.4 \; \text {fps}/19.6\;\text {fps}/21.0\;\text {fps}$ respectively. We see that except for Mate9, the frequency of all the other two is around $20\;\text {fps}$, which is close to the realtime frequency of $24\;\text {fps}$. This near-realtime speed can guarantee the fluency of the app and good user experience, which can be beneficial to put forward the application worldwide. For Mate9, we see there is still some gap to the realtime speed, which indicates the challenge in the situations with extreme low computation cost, and we will make our effort to achieve the goal in our future study. Figure 8 shows some screenshots of the application interface on the above three phones with their true resolution ratio. We see the herbs can be correctly recognized in all the phones with a high confidence, which provides an effective tool to recognize herb categories and improve the quality control of the herbal medicine.

### Large-scale validation

To validate the effectiveness of the proposed network compression on a large-scale dataset, we further evaluate on the ImageNet with 1000 categories and 1.2 million images^51^. This dataset is used for general image classification task, thus it is highly related to our task. Different from the above setup in network selection, we choose the ResNet-50^15^ as the small DNN, as the capacity of MobileNet-V2 can no longer hold this large-scale case. Correspondingly, we change the large DNN to the powerful ResNeXt-101^16^.

Table 3 shows the top1 and top5 classification accuracy of the large and small DNN throughout the whole compression process, and we have three main observations. Firstly, in the network pre-train step, the small DNN only gets the top1/top5 accuracy of $75.3\%/92.2\%$, while the large DNN obtains the top1/top5 accuracy of $79.2\%/94.3\%$. The accuracy gap between them is expected as the large DNN has a network depth of 101 layers and 2*X* parameters than the small DNN, thus its recognition ability is stronger. Then, in the network transfer step, we observe that the small DNN with network transfer has achieved encouraging results by being very competitive to the large DNN, i.e. the top1 accuracy difference is within $1\%$ ($78.4\%$ vs $79.2\%$) and the top5 accuracy is even higher than the large DNN ($94.5\%$ vs $94.3\%$). This result is similar to what we have observed in the small-scale herb image dataset, which indicates the network transfer can fully discover the potential of learning ability and also demonstrates the effectiveness and generality of the network transfer. Finally, in the network cut step, we show that by cutting $50\% \; (\alpha =0.5$) parameters of the small DNN, the compressed small DNN can still be competitive to the original small DNN, i.e. the top1 accuracy difference is $0.5\%$ ($74.8\%$ vs $75.3\%$) and the top5 accuracy is higher ($92.4\%$ vs $92.2\%$). This result means a lot in the industry as with only half the network parameters, the compressed small DNN can achieve the comparable accuracy to the original small DNN, but runs much faster in efficiency with only half the network size in memory cost. The above results have validated the effectiveness of the proposed network compression method in the large-scale situation.

## Discussion

In this part, we will discuss the possible future work of deep learning algorithms on herb image classification.

### Neural architecture search

A network structure with efficient design plays a vital role in herb image classification in resource-limited settings. In light of this, we choose mobilenets as the small DNN because of the comparable accuracy and higher efficiency with fewer parameters. Though some efficient structures have shown promising results^33–37^, there is still plenty of room for improvement, as most of them are manually designed. The Neural Architecture Search (NAS), which is a hot research topic in recent years, can automatically search for the best structure that fits the training task. By setting some targets such as comparable accuracy with fewer parameters and higher efficiency, NAS will search for the best design that can maximize the targets. Besides, NAS can contribute to the network compression. In the network cut step where a fixed cutting ratio has been used for all layers, NAS can search for the best cutting ratio in each layer to jointly give the best trade-off between accuracy and efficiency. There’s reason to believe that NAS is the future of herb image classification, as it can largely reduce human effort on network design and hyper-parameter tuning, especially in resource-limited settings with strict requirements on accuracy and efficiency.

**Table 3:** Main results for evaluation on the ImageNet dataset.

DNN strategy	Top1 acc (%)	Top5 acc (%)
large DNN (ResNeXt-101)	79.2	94.3
small DNN (ResNet-50)	75.3	92.2
small DNN + transfer	78.4	94.5
small DNN + transfer + cut ($\alpha =0.5$)	74.8	92.4

### Public dataset construction and automatic annotation

Public datasets have a place in creating new techniques all over the world. As in the case for the large-scale ImageNet dataset^51^ (1000 categories, 1.2 million images), plenty of new methods have been proposed over the past 10 years since the ImageNet competition in 2012. However, in herb image classification, there are mainly two datasets of small scale: one is the dataset used in this paper with 95 categories and 5600+ images^24^, and the other one is the unpublished dataset with 315 categories and 31,000+ images^27^. Therefore, it is necessary to establish a large-scale public dataset to promote the development of herb image classification. Another key problem is how to give annotations to all images when the dataset is ready. Different from the general object classification task wherein objects are easily classified, annotating herb categories needs professionals with expert knowledge and costs lots of time and efforts. Thus, it is also necessary to develop automatic annotation algorithms to reduce human efforts. Actually, we have been working on solving these two problems over the past several months, and we will release a large-scale dataset with automatic annotation in the near future.

### Transfer learning and domain collaboration

In general image classification, pre-training on ImageNet has been successfully transferred to many down-stream tasks such as object detection^53^, image segmentation^52^ and image retrieval. Similarly, once the large-scale herb image dataset is ready, pre-training on it can largely boost its down-stream tasks such as herb detection and herb segmentation, and some similar tasks such as plant image classification^25,28^ and flower image classification will also benefit a lot. We believe various transfer learning methods will be proposed in the near future to extend the influence of herb image classification in the research community. Another related problem is how to jointly use the herb image data from different domains, namely, domain collaboration. For example, some herb images are taken in the laboratory with clean background and high-resolution, while other images are downloaded from Internet with cluttered background and low resolution. Due to the domain difference, directly combining them in training will affect the classification accuracy in each domain. Therefore, it is of significance to work out an effective domain collaboration method in order to maximize the data utilization of herb images.

## Conclusion

In this paper, we present a deep learning-driven smartphone application for efficient and robust herb recognition. The App first takes and pre-processes an image of testing herbs with some data augmentation, then it runs the DNN computation based on the compressed DNN generated by the network compression algorithm, and finally it gives candidate herb categories. The whole procedure is performed entirely on a single common smartphone, which can serve perfectly for resource-limited situations.

The proposed network compression method shows promising performance. Firstly, with only half the parameter size of the original small DNN, the compressed small DNN has achieved the comparable accuracy, which is therefore considered satisfactory for usage in resource-limited settings. Secondly, we tested the efficiency on three common smartphones, and two of them can perform at the near-realtime speed, which demonstrates the efficacy of the network compression algorithm and indicates that the App can provide a great user experience with fast response and low power consumption.

To obtain a small DNN with high accuracy and efficiency, we have proposed a network compression algorithm, which contains three main steps. Firstly, we pre-train the small DNN on a large-scale dataset to learn general representation and give stable initialization, which can achieve better generality and speed up the training. Then, to satisfy the accuracy requirement, the network transfer was applied to achieve the recognition ability transformation from the large DNN to the small one. Finally, we use the network cut to achieve the best trade-off between efficiency and accuracy.

This study aims to encourage the implementation of herb recognition in resource-limited dispensaries, hospitals and laboratories where expensive computation hardware cannot be supported. Firstly, instead of competing with large DNNs with powerful hardware, the App place more stress on high degree of automation, with low cost and high efficiency. Secondly, rather than compete with the manual herb recognition, through reducing requirements in chemical devices and expert resources, the App is designed to provide confident candidate decisions to assist the manual recognition. Finally, with the integration of herb recognition and a single smartphone, we hope this study can fill the digital gap between the public and herbal medicine so as to increase the accessibility of herbal medicine worldwide.

## Data Availability

The herb image dataset used in this paper is available on google drive with the shared link https://drive.google.com/drive/folders/10BGJYMcrSsbonilPVwkC8eBONss-TCw9?usp=sharing.

## Code availability

The network compression algorithm described in this paper is distributed as an open-source project at https://github.com/bucmsunxin/herb_image_compress. Meanwhile, we also release the mobile application on an open-source repository at https://github.com/bucmsunxin/herb_image_recognition_app.
